# Repetitive Behaviours in Autistic and Non-Autistic Adults: Associations with Sensory Sensitivity and Impact on Self-Efficacy

**DOI:** 10.1007/s10803-023-06133-0

**Published:** 2023-09-26

**Authors:** Gabrielle Nwaordu, Rebecca A. Charlton

**Affiliations:** Department of Psychology, Goldsmiths University of London, New Cross, London, SE14 6NW UK

**Keywords:** Adulthood, Coping, Repetitive behaviours, Sensory sensitivities, Self-efficacy, Stimming

## Abstract

Purpose: Restricted and repetitive behaviours are a core feature of autism diagnoses but have not been widely studied in adulthood. This study examined the rates of and associations between repetitive behaviours and sensory sensitivity in autistic and non-autistic adults; and whether repetitive behaviours described as “stimming” impacted coping with difficulties (self-efficacy). Methods: Diagnosed autistic (n = 182), undiagnosed autistic (n = 163) and non-autistic (n = 146) adults completed online measures of repetitive behaviours, sensory sensitivity, and self-efficacy for when able and not able to stim. Results: Repetitive behaviours and sensory sensitivity correlated significantly in each group, although ratings were higher in autistic compared to non-autistic groups. When people were able to stim, no differences between the groups were observed on self-efficacy ratings. However when unable to stim, autistic people reported lower self-efficacy than non-autistic people. Conclusions: Results suggest that repetitive behaviours are significantly associate with sensory sensitivities. Rather than repetitive behaviours being viewed as negative, stimming was associated with increased self-efficacy. Results suggest that stimming may have beneficial effects. Further work is needed to better understand how repetitive behaviours and stimming manifest in adulthood, how they change over time and their effects for autistic adults.

## Introduction

Restricted and repetitive patterns of behaviour and sensory sensitivities are core features of the diagnostic criteria for Autism Spectrum Conditions (ASC; American Psychiatric Association, 2013). Repetitive behaviours are characterised as including stereotyped repetitive sensory-motor behaviours (RSMB) and insistence on sameness (IS) which can include restricted interests (Barrett et al., 2018; Leekam et al., 2011). Under ASC diagnostic criteria unusual sensory responsiveness (henceforth sensory sensitivities) is also included as a feature of repetitive behaviours. Sensory sensitivities can manifest as hyper- or hypo-sensitivity and within any (or multiple) sensory domains, with hyper- and hypo-sensitivity both being possible for an individual (Robertson & Simmons, 2013). It has been suggested that repetitive behaviours may help to distract an individual from an overwhelming sensory environment (hypersensitivity), act as a coping mechanism or provide stimulation in the face of hyposensitivity to the environment (Joyce et al., 2017; Kapp et al., 2019; Robertson & Simmons, 2015). Repetitive behaviours are not unique to autism, but are more common among autistic than non-autistic individuals (Harrop et al., 2014; Leekam et al., 2011; Schulz & Stevenson, 2018). It is worth noting that studies of repetitive behaviour among non-autistic people without co-occurring conditions are sparse (Robertson & Simmons, 2013), although note that repetitive behaviours are common among children with other developmental or sensory impairments (Dammeyer, 2014; Hartshorne et al., 2005; Ivy & Ledford, 2022). In this paper we will use the term “stimming” to describe repetitive actions and behaviours that participants categorise as stimming (see Methods for more detail). We will also use “repetitive behaviours” to describe a broad range of RSMB and IS when these terms are used by measures and in previous studies.

Among autistic children, many studies have explored repetitive behaviours, sensory sensitivities and associations between these variables, but few studies have explored these variables in adulthood (Harrop et al., 2014; South et al., 2005). Some (but not all) studies have identified fewer repetitive behaviours with age, measured by examining cross-sectional differences between autistic people in different age-groups (Barrett et al., 2018; Leekam et al., 2011). Such results alongside some anecdotal reports have been interpreted as a reduction in repetitive behaviours with increasing age. However RSMB remain commonly reported by autistic adolescents and adults (Charlton et al., 2021; Kapp et al., 2019). One study measured whether repetitive behaviours changed with age, by examining current and retrospective (ever in the lifetime) informant reports for autistic adults (Chowdhury et al., 2010). Repetitive behaviours reduced with age in five of six domains (stereotyped, self-injurious, compulsive, ritualistic, and sameness behaviours) but did not reduce in restricted behaviours. It remains unclear whether repetitive behaviours, the reason for such behaviours, and the pattern of associations with other variables are the same in childhood and adulthood.

One explanation for some of the discrepancies in frequency of repetitive behaviours may relate to whether studies use self or informant report. A study of autistic adolescent and young adults found very poor agreement between parental-report and self-report ratings of repetitive behaviours (Joyce et al., 2017). Although the source of the disagreement was not investigated, examples of responses suggest that stimming was reported more frequently by self-report than parent-report. In contrast, IS was reported as higher by parent-report than by the self. One explanation for these discrepancies may be that autistic people hide stims or use substitute (more socially acceptable) stims. Previous studies suggest that autistic people often do not stim the way they want to due to social pressure including from close family members, therefore discrepancies may be due self-report reflecting own behaviour and other-report reflecting observed behaviours (Charlton et al., 2021; Dachez & Ndobo, 2018; Kapp et al., 2019).

Informant-report versus self-reported may influence the perception of repetitive behaviours as positive or negative. In studies of autistic children, stimming is often described as negative and something to be eliminated (Leekam et al., 2011). In contrast, recent studies with autistic adults describe stimming as largely positive (Charlton et al., 2021; Joyce et al., 2017; Kapp et al., 2019; Stewart, 2015). Although stimming is often described as pleasant for its own sake, many autistic people also describe stimming as a coping mechanism that helps to reduce anxiety, organise thoughts and distract from environmental stressors (Charlton et al., 2021; Joyce et al., 2017; Kapp et al., 2019; Manor-Binyamini & Schreiber-Divon, 2019; Stewart, 2015). This benefit occurs despite autistic adults being discouraged from stimming, told explicitly not to stim, and often modifying or hiding stims in order to avoid criticism (Charlton et al., 2021; Dachez & Ndobo, 2018; Kapp et al., 2019). Studies suggest that autistic people frequently supress their preferred stims and these substitute stims may be less effective as coping mechanisms (Hull et al., 2017; Livingston et al., 2018). To our knowledge no study has yet examined the impact of stimming on autistic and non-autistic adults’ ability to cope with everyday difficulties. Given that stress levels are reported as higher and coping lower among autistic compared to non-autistic adults, understanding whether stimming can support self-efficacy may have a significant impact on perceptions of stimming and the everyday lives of autistic adults (Hirvikoski & Blomqvist, 2014).

Although qualitative studies suggest that repetitive behaviours may help autistic people cope with the sensory environment, few studies have quantitatively examined the potential positive effects of stimming (MacLennan et al., 2022). One’s own belief about ability to cope with a task or situation, to manage everyday difficulties and be successful is called self-efficacy (Schwartzer & Jerusalem, 1995). Programmes are attempting to improve self-efficacy (mostly around education and employment) for autistic young adults, but to our knowledge no studies have examined whether stimming or supressing stims influences self-efficacy (Burke et al., 2021; Shattuck et al., 2014; Ward & Esposito, 2019). Understanding whether stimming can improve self-efficacy especially in challenging sensory environments is important for both understanding and developing interventions.

Positive and negative sensory sensitivities are commonly reported among autistic children and adults, and are one issue that contributes to environmental stressors (Boyd, McBee, Holtzclaw, Baranek, & Bodfish, 2009; Joyce et al., 2017; Robertson & Simmons, 2015). When sensory stimuli is experienced as negative, it can cause both physical and emotional distress (Charlton et al., 2021; Robertson & Simmons, 2015). Few studies have explored the relationship between sensory sensitivities and repetitive behaviours among autistic adults, but strong associations are observed between these variables (Hwang et al., 2020; Kargas et al., 2015; Moore et al., 2022). Among autistic adults, individuals with enhanced auditory processing (which may lead to overstimulation) demonstrated more repetitive behaviours on the Autism Diagnostic Observation Schedule (Kargas et al., 2015). It has been suggested that variability in sensory processing may contribute to sensory sensitivities and be a factor in the development and maintenance of repetitive behaviours (Haigh, 2018). Recent studies have found significant correlations between self-report sensory sensitivities and repetitive behaviours (both RSMB and IS) among autistic and non-autistic adults’ (Hwang et al., 2020; Moore et al., 2022). Mediation analyses demonstrated direct effects between sensory sensitivities and repetitive behaviours (as well as indirect effects through alexithymia, insistence on sameness and anxiety; Moore et al., 2022). Studies have identified the same pattern of correlations between repetitive behaviours and sensory sensitivity among autistic and non-autistic adults, suggesting this association is not exclusive to autism (Hwang et al., 2020; Schulz & Stevenson, 2018). Results so far suggest strong associations between repetitive behaviours and sensory sensitivities among autistic adults, with non-autistic adults demonstrating weaker but similar patterns of associations between these variables.

The aims of this study are to examine the associations between repetitive behaviours and sensory sensitivity among autistic and non-autistic adults, and to explore the impact of stimming on self-efficacy.

### Hypotheses

We hypothesise that (1) diagnosed and undiagnosed autistic adults will report more repetitive behaviours and higher levels of sensory sensitivity compared to non-autistic adults; (2) a significant correlation will be observed between repetitive behaviours and sensory sensitivity, and (3) that the magnitude of the correlation will be greater among autistic than non-autistic adults. We further hypothesise (4) that stimming will increase self-efficacy compared to when individuals cannot stim, and (5) that substitute stims will be less helpful than preferred stims.

## Methods

### Procedure

Autistic and non-autistic adults were recruited to participate in this study through social media, in-person and online support groups for autistic individuals including an autistic mothers’ groups (people who were both autistic and mothers), and general advertising and promotion of the study. The study was hosted on an online platform Qualtrics (Provo, UT, USA; https://www.qualtrics.com). Interested participants were presented with an online information sheet, General Data Protection Regulation information, and were asked to provide informed consent. Questionnaires were then completed online (see Materials section). All research was carried out per the Declaration of Helsinki and in keeping with General Data Protection Act, 2018. Ethical approval was awarded by Goldsmiths University of London Research Ethics Committee.

### Participants

The study was started by 887 people; 322 people provided no data or did not give consent to participate. A further 74 people were excluded due to not completing the first questionnaire, not providing demographic information or being aged between 16 and 18 years old. Data was available for at least one questionnaire for 491 adults. As part of the demographic information participants reported whether they have a formal diagnosis of an autism spectrum condition and when they received their diagnosis (Diagnosed Autistic group, DA, n = 182), self-identified as autistic, suspected they were autistic or were currently seeking an autism diagnosis (Undiagnosed Autistic group, UA, n = 163), or were not autistic (Non-Autistic group, NA, n = 146). See Table 1 for full demographic information. Due to the method of recruitment (via support groups for independent autistic people and social media) and task demands (following survey links, reading information and instructions and completing the survey), we expect that most participants will have intellectual ability within the average range, although ability was not measured.

**Table 1:** Mean and standard deviations for demographic information and questionnaire scores, by group

	Diagnosed Autistic N = 182	Undiagnosed Autistic N = 163	Non-Autistic N = 146	Group differences
Age	36.35 (9.55) Range = 18–60	37.65 (8.18) Range = 18–62	37.53 (7.84) Range = 21–58	F = 1.20, p = .302 DA = UA = NA
Sex (male, female, non-binary/other, prefer not to say)	17, 144, 20, 1	4, 149, 8, 2	9, 135, 2, 0	*X*^2^ = 21.65, p < .001
RBQ-2 A Total ^a^	2.20 (0.38)	2.15 (0.36)	1.49 (0.38)	F = 180.14, p < .001 DA = UA > NA
RBQ-2 A RSMB sub-scale	1.26 (0.26)	1.22 (0.26)	0.83 (0.23)	F = 134.83, p < .001 DA = UA > NA
RBQ-2 A IS sub-scale	3.13 (0.58)	3.07 (0.55)	2.11 (0.59)	F = 155.34, p < .001 DA = UA > NA
GSQ Total ^b^	82.73 (23.41) N = 171	80.96 (20.00) N = 155	41.05 (21.00) N = 132	F = 167.93, p < .001 DA = UA > NA
GSQ Hypersensitivity	47.56 (14.01)	46.75 (11.77)	24.30 (11.98)	F = 152.28, p < .001 DA = UA > NA
GSQ Hyposensitivity	35.16 (11.07)	34.22 (10.14)	16.75 (10.20)	F = 137.61, p < .001 DA = UA > NA
Participants who said “yes” they stim	n = 121 (66% of DA group)	n = 106 (67% of UA group)	n = 29 (20% of NA group)	
Self-efficacy when Stimming ^c^	10.76 (2.56)	10.73 (2.42)	10.24 (2.36)	F = 0.230, p = .795 DA = UA = NA
Self-efficacy when NOT Stimming	6.36 (3.29)	6.34 (3.39)	8.07 (3.94)	F = 4.06, p = .019 DA = UA < NA
Paired sample t-test for difference in self-efficacy when stimming	t = 14.51, p < .001	t = 12.70, p < .001	t = 3.78, p = .001	

*Note.*^a^ RBG-2 A = The Adult Repetitive Behaviour Questionnaire-2; RSMB = Repetitive Sensory Motor Behaviour; IS = Insistence on Sameness; Normative data from Barrett et al. (2015) for RBQ-2 A: Autistic group, Total, M = 1.84, sd = 0.45; RSMB, M = 1.64, sd = 0.47; IS, M = 2.04, sd = 0.55; Comparison group, Total, M = 1.25, sd = 0.19; RSMB, M = 1.20, sd = 0.24; IS, M = 1.29, sd = 0.25. ^b^ GSQ = Glasgow Sensory Questionnaire. ^c^ Self-efficacy data is reported only for individuals who responded to both when stimming and when not stimming

Individuals who identified as autistic but had not received a diagnosis completed the Ritvo Autism Asperger Diagnostic Scale-Revised (RAADS-R; Ritvo et al., 2011), to assess whether individuals were likely to be autistic. The RAADS comprises 14 statements which individuals rate according to a four-point Likert scale (0 = Never true; 1 = True only when I was younger than 16; 2 = True only now; 3 = True now and when I was young). Scores of 14 or higher are classified as the cut-off for having suspected autism (Sensitivity = 97%; Specificity = 95%). Scores on the RAADS demonstrated acceptable internal consistency (Cronbach’s alpha: α = 0.757). All individuals in the UA group scored above the cut-off for suspected autism (Range = 17–42; Mean = 33.34, SD = 6.47).

### Materials

All participants provided demographic information and then completed The Adult Repetitive Behaviours Questionnaire-2 and the Glasgow Sensory Questionnaire.

The Adult Repetitive Behaviours Questionnaire-2 (RBQ-2 A) is a self-report measure that produces a total overall score, and two sub-scales measuring Repetitive Sensory Motor Behaviours (RSMB) and Insistence on Sameness (IS) (Barrett et al., 2018). The RBQ-2 A total comprises 20 questions rated on a 1–3 Likert scale (Never or rarely; one or more times daily; 15 or more times daily). The RSMB sub-scale is based on responses to six questions, and the IS subscale is based on responses to 11 questions. All scores are reported as mean scores. All scores demonstrated acceptable internal consistency (Cronbach’s alpha: Total, α = 0.936; RSMB, α = 0.791; IS, α = 0.892).

The Glasgow Sensory Questionnaire (GSQ) measures hyper- and hypo-sensitivity in seven sensory domains (Robertson & Simmons, 2013). Forty-two items are rated on a 0–4 Likert scale (Never, Rarely, Sometimes, Often, Always). The hyper- and hypo-sensitivity sub-scales are calculated based on 21 items each. All scores demonstrated acceptable internal consistency (Cronbach’s alpha: Total, α = 0.951; Hypersensitivity, α = 0.930; Hyposensitivity, α = 0.887).

Participants were asked if they “stimmed” (“Do you do any stims*, or repetitive movements? *Stimming normally describes the way people move sometimes in a repetitive manor, for example finger clicking, chewing (pen lids), rocking on a chair or spinning. There are also things often described as visual stims, these may include watching intently at light refraction off water or specs of dust in the air. Audible stims often include whistling, humming, clapping etc.”). Only participants who answered “yes” to this question (66% of DA; 67% of UA; 20% NA groups) completed subsequent questions. Participants who reported that they did stim were asked to rate their self-efficacy on five questions in two scenarios: when they were and were not able to perform stims or repetitive behaviours. Items asked about how able individuals felt able to (1) solve problems, (2) stick to aims and accomplish goals, (3) deal with unexpected events, (4) find solutions to difficulties, (5) handle whatever happens. Each item was rated on a 0–3 point Likert scale (Not at all true, Hardly true, Moderately true, Exactly true). Items were summed to produce a self-efficacy score for when able to stim and when not able to stim. Total scores demonstrated acceptable internal consistency (Cronbach’s alpha: Self-efficacy when able to “stim”, α = 0.854; Self-efficacy when not able to “stim”, α = 0.873).

Participants who reported stimming were asked additional questions. They were asked whether stimming was helpful in managing sensory experiences (rated on a 7-point Likert scale, from 1 = Extremely useless to 7 = Extremely helpful); whether they had changed stims to be socially acceptable (yes or no), and how helpful substitute stims were if they were used (rated on the same 7-point Likert scale as above).

### Community Involvement Statement

The research team includes an autistic researcher who designed the current study. No other community members were involved in the study design.

### Statistical Analysis

Group differences were assessed using Chi-square analysis, univariate and repeated measures ANOVAs. Post-hoc analyses were explored using independent and paired-sample t-tests. Pearson correlations were performed to assess associations between variables of interest. Due to participant characteristics (wide age range, high proportion of women), post-hoc correlations to examine the relationship between age and variables of interest were performed, and analyses were repeated for women only.

## Results

### Demographic Information

#### Group Differences

There were no significant differences between the groups in age of participants. A significant difference in gender was noted between the groups, with more males and more non-binary individuals in the DA group. See Table 1 for results.

### Variables of Interest

#### Group Differences

Significant group differences were observed on total and sub-scale measures for both self-report repetitive behaviours (RBQ-2 A) and sensory sensitivity (GSQ). For all measures, there were no significant differences between DA and UA groups. Both autism groups (DA and UA) reported significantly higher scores than the NA group. See Table 1 for results.

#### Correlational Analyses

**Fig. 1:**
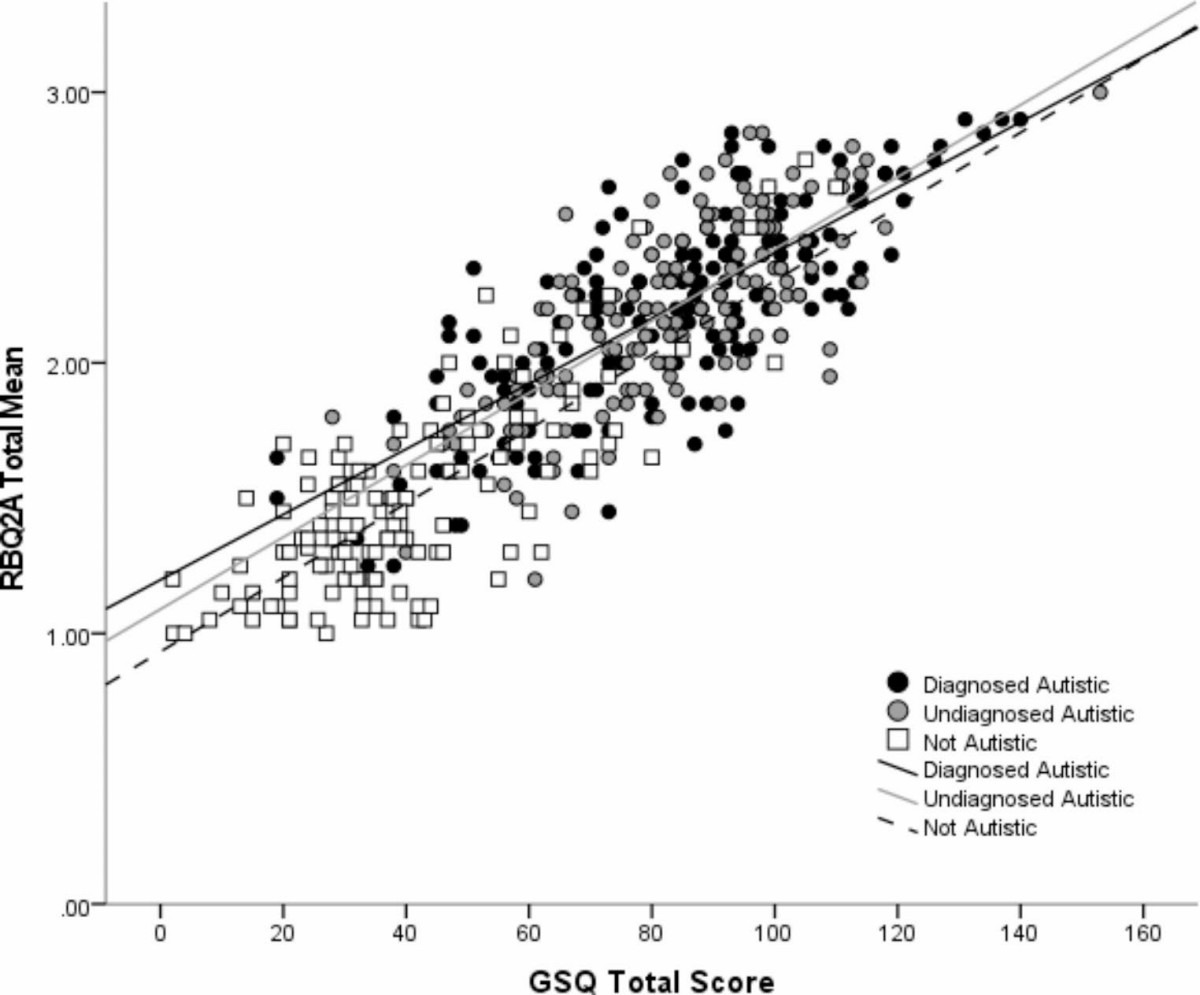
Scatterplot showing correlation between total scores on The Adult Repetitive Behaviour Questionnaire-2 (RBG-2 A) and Glasgow Sensory Questionnaire (GSQ)

Examining the whole sample, highly significant correlations were observed between all measures of repetitive behaviour and sensory sensitivity. RSMB and IS correlated significantly with both hypersensitivity and hyposensitivity. The same pattern of correlations was observed for all three groups. No significant differences in the magnitude of correlations were observed. See Table 2 for all correlation results and Fig. 1 for the association between total scores on the RBQ-2 A and the GSQ.

**Table 2:** Correlations between repetitive behaviour and sensory sensitivities, by group

	GSQ Total ^b^	GSQ Hypersensitivity	GSQ Hyposensitivity
		**Whole Sample (n = 458)**	
RBQ-2 A Total ^a^	*r = .867, p < .001*	*r = .812, p < .001*	*r = .853, p < .001*
RBQ-2 A RSMB sub-scale	*r = .792, p < .001*	*r = .728, p < .001*	*r = .798, p < .001*
RBQ-2 A IS sub-scale	*r = .818, p < .001*	*r = .776, p < .001*	*r = .793, p < .001*
		**Diagnosed Autistic (n = 171)**	
RBQ-2 A Total ^a^	*r = .770, p < .001*	*r = .703, p < .001*	*r = .739, p < .001*
RBQ-2 A RSMB sub-scale	*r = .651, p < .001*	*r = .556, p < .001*	*r = .673, p < .001*
RBQ-2 A IS sub-scale	*r = .698, p < .001*	*r = .661, p < .001*	*r = .640, p < .001*
		**Undiagnosed Autistic (n = 155)**	
RBQ-2 A Total ^a^	*r = .754, p < .001*	*r = .640, p < .001*	*r = .744, p < .001*
RBQ-2 A RSMB sub-scale	*r = .665, p < .001*	*r = .558, p < .001*	*r = .666, p < .001*
RBQ-2 A IS sub-scale	*r = .650, p < .001*	*r = .560, p < .001*	*r = .632, p < .001*
		**Non-Autistic (n = 132)**	
RBQ-2 A Total ^a^	*r = .783, p < .001*	*r = .694, p < .001*	*r = .798, p < .001*
RBQ-2 A RSMB sub-scale	*r = .695, p < .001*	*r = .599, p < .001*	*r = .728, p < .001*
RBQ-2 A IS sub-scale	*r = .736, p < .001*	*r = .664, p < .001*	*r = .737, p < .001*

*Note.*^a^ RBG-2 A = The Adult Repetitive Behaviour Questionnaire-2; RSMB = Repetitive Sensory Motor Behaviour; IS = Insistence on Sameness; ^b^ GSQ = Glasgow Sensory Questionnaire. Italics indicates correlation was significant at the 5% level

#### Impact of Stimming on Self-Efficacy

**Fig. 2:**
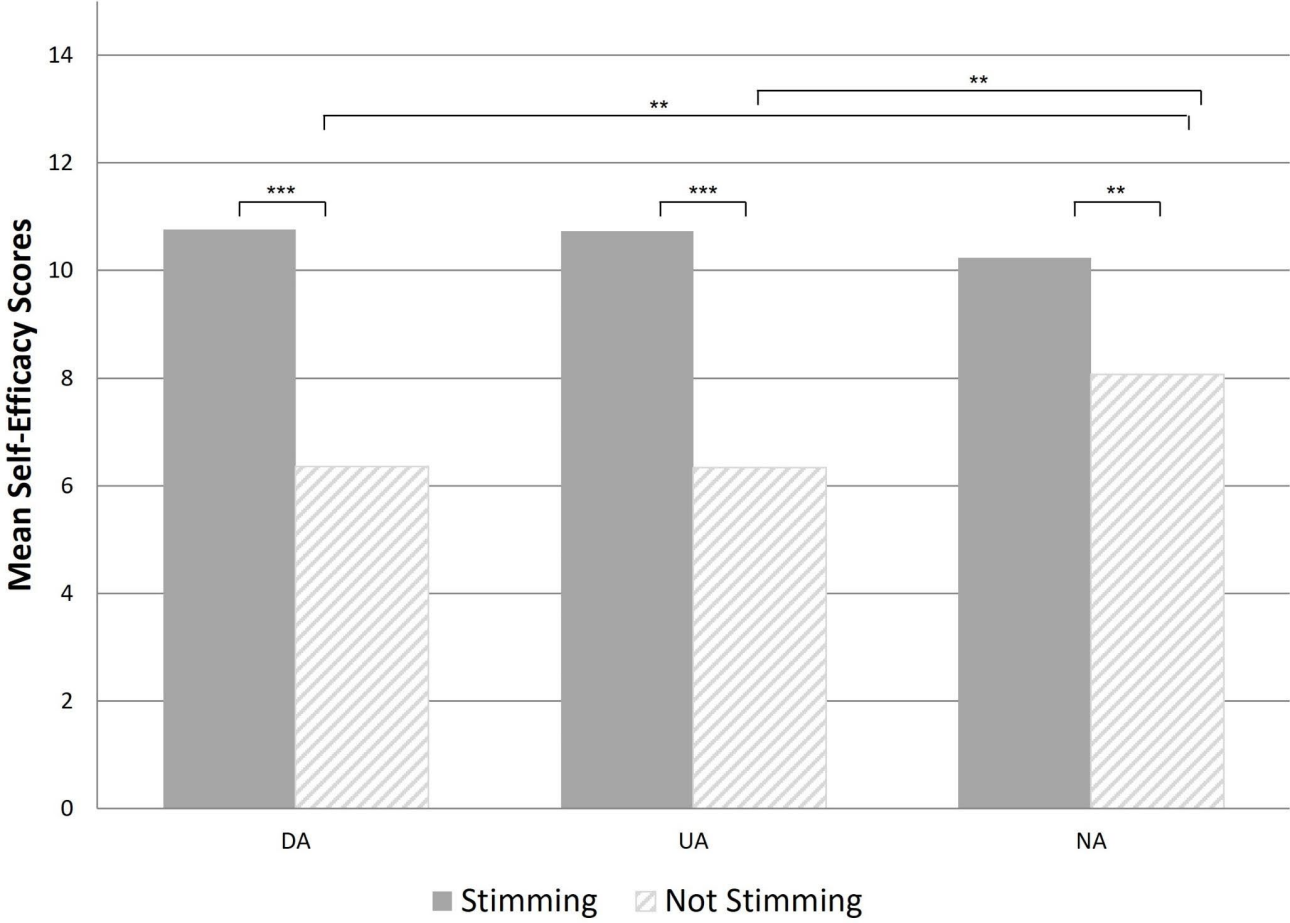
Impact of Stimming versus Not Stimming on Self-Efficacy ratings, by Group *Note.* DA = Diagnosed Autistic; UA = Undiagnosed Autistic; NA = Anon-Autistic; *** p < .001; **p < .01

A repeated measures ANOVA was performed to assess the impact of stimming on self-efficacy, between the groups. A significant effect of stimming was observed with higher self-efficacy reported when people were able to stim, compared to when they could not stim (F = 198.63, p < .001). No significant effect of group was observed (F = 0.801, p = .450). A significant group by stimming interaction was observed (F = 5.48, p = .005). Post-hoc t-tests reveal a significant difference between stimming and not-stimming for all three groups. Post-hoc ANOVA revealed no differences between the groups when people were able to stim. However, when not able to stim, the two autism groups reported significantly lower self-efficacy than the non-autistic group. See Table 1; Fig. 2 for results.

#### Preferred Versus Substitute Stims

A repeated measures ANOVA compared the usefulness of preferred versus substitute stims and explored the effect of group. There was a significant effect of stim-type, with preferred stims being more helpful than substitute stims (F = 58.21, p < .001). There was no significant effect of group (F = 0.128, p = .880) and no significant interaction (F = 0.426, p = .654). For all groups substitute stims were less effective than preferred stims.

### Post-hoc Analyses

#### Correlations with Age

The wide age-range included in the study allowed age-effects to be explored within each group. Age was not strongly associated with scores on either the RBQ-2 A or the GSQ. Only two significant correlations were observed both in the diagnosed autistic group, where age correlated significantly with RSMB on the RBQ-2 A (r=-.157, p = .034) and hyposensitivity on the GSQ (r=-.213, p = .005) indicating fewer reports of RSMB and hyposensitivity with older age. Fisher’s r was used to assess whether the groups differed in the magnitude of the correlations; no significant differences between the correlations were observed. See Table 3 for correlations with age for the whole sample and by group.

**Table 3:** Correlations between age and RBQ and GSQ scores, by group

	Age			
	Whole Sample	Diagnosed Autistic	Undiagnosed Autistic	Non-Autistic
RBQ-2 A Total	*r=-.095, p = .035*	r=-.097, p = .194	r=-.091, p = .246	r=-.103, p = .216
RBQ-2 A RSMB sub-scale	*r=-.153, p = .001*	*r=-.157, p = .034*	r=-.108, p = .171	r=-.146, p = .079
RBQ-2 A IS sub-scale	r=.-0.0789 p = .080	r=.-0.089 p = .233	r=-.079, p = .314	r=-.066, p = .428
GSQ Total	*r=-.093, p = .046*	r=-.129, p = .092	r=-.007, p = .929	r=-.102, p = .245
GSQ Hypersensitivity	r=-.048, p = .301	r=-.048, p = .533	r = .055, p = .499	r=-.088, p = .313
GSQ Hyposensitivity	*r=-.140, p = .003*	*r=-.213, p = .005*	r=-.078, p = .336	r=-.106, p = .226

*Note.* RBG-2 A = The Adult Repetitive Behaviour Questionnaire-2; RSMB = Repetitive Sensory Motor Behaviour; IS = Insistence on Sameness; GSQ = Glasgow Sensory Questionnaire. Italics indicates correlation was significant at the 5% level. Fisher’s r was used to assess whether differences between the correlations were significant. No significant differences between the correlations were observed

#### Women-Only Analysis

Given that the sample was largely female (87%), the analyses were repeated including only female participants. Since only a small proportion of the sample are male or non-binary (6% each but with smaller percentages across groups), comparisons based on sex were not performed. For all analysis the pattern of results remained the same for the female only analyses (data not reported).

## Discussion

This study examined self-report ratings of repetitive behaviours and sensory sensitivities among autistic and non-autistic adults. Diagnosed and undiagnosed autistic adults reported more repetitive behaviour and higher sensory sensitivity than non-autistic adults. Results showed the same pattern for both total scores and sub-scales (repetitive sensory motor behaviours and insistence on sameness) for repetitive behaviour, and hyper- and hypo-sensitivity for sensory sensitivity. These higher rates of both repetitive behaviour and sensory sensitivity are in keeping with previous studies and demonstrate that these behaviours continue into adulthood for many autistic people (Bodfish, Symons, Parker, & Lewis, 2000; Hwang et al., 2020). Within the current study the undiagnosed autism group show the same pattern of results as the diagnosed autism group. It is worth noting that the undiagnosed autism group meet the criteria for suspected autism according to an autism screening measure (Ritvo et al., 2011). Results suggest that some individuals in the undiagnosed autism groups may be eligible for an autism diagnosis although they have not yet received one, as such they may represent an understudied population.

Results in this study support the hypothesis that a significant correlation would be observed between self-reported repetitive behaviours and sensory sensitivity. The same pattern of associations was observed for both repetitive sensory motor behaviours and insistence on sameness with hyper- and hypo-sensitivity. We also hypothesised that the magnitude of the correlations would be greater in the autistic compared to non-autistic groups, and this hypothesis was not supported. Across all three groups (diagnosed autistic, undiagnosed autistic and non-autistic) correlations between the total and sub-scale scores were moderate to high, and no significant differences between the pattern of correlations were observed. The robust correlations between repetitive behaviours and sensory sensitivity in the autistic groups are in keeping with previous studies of children and adults (Hwang et al., 2020; Schulz & Stevenson, 2018). The magnitude of correlations observed in the current study for the non-autistic group was higher than those observed previously in non-autistic children (r = .44; Schulz & Stevenson, 2018) and university students (r = .3; Schulz & Stevenson, 2019), but at a similar magnitude to a study including non-autistic adults across the lifespan (r = .59; Hwang et al., 2020). These results suggest a strong association between repetitive behaviours and sensory sensitivity not only among autistic individuals but also in the general population. Although the recruitment approach in the current study may have over-sampled individuals who identify as having repetitive behaviours and sensory sensitivities, these traits may be commonly expressed (although at a lower level) among non-autistic adults.

We further examined the effect of stimming on individuals’ self-efficacy. Results suggest that stimming increased people’s perception of their ability to cope with everyday difficulties. Although fewer people in the non-autistic group reported stimming compared to the autistic groups (20% in NA compared to 66% in DA and 67% in UA groups), for individuals who reported stimming the behaviour had a significant positive impact on self-efficacy. When able to stim, no group differences were reported in self-efficacy. This is an important finding as it indicates that when they feel free to stim, autistic people report ability to cope at the same level as non-autistic people. This result supports findings from qualitative studies suggesting that stimming can be beneficial and help people to cope with challenges in the environment (Joyce et al., 2017; Kapp et al., 2019; Robertson & Simmons, 2015). Furthermore, the impact of not being able to “stim” was reported to be more detrimental to autistic groups compared to the non-autistic group. This suggests that whilst not being able to stim reduces self-efficacy for both autistic and non-autistic adults, being unable to stim is particularly disadvantageous for autistic adults. Results demonstrated that substitute stims (stims that have been changed to be more socially acceptable), were reported to be less beneficial than preferred stims for all groups. Results suggest that social pressures on autistic people to supress or alter stims, may reduce their ability to cope with day-to-day challenges. These findings are in keeping with previous qualitative studies which report that autistic people frequently supress stims and repetitive behaviours to be more socially acceptable and that doing so was detrimental to the individual (Cook et al., 2021; Hull et al., 2017; Livingston et al., 2018; Manor-Binyamini & Schreiber-Divon, 2019). Previous studies on masking (including masking stims) suggest that masking is exhausting, stressful and associated with high levels of anxiety (Bradley et al., 2021; Livingston et al., 2018). The current findings are in keeping with previous literature and suggests that both not being able to stim and substituting stims are detrimental to the ability to cope, which may be partly due to both the effort of masking and the loss of the benefits of stimming how one prefers. To our knowledge no other study has measured self-report self-efficacy when able or not able to stim, or asked people to rate the efficacy of preferred versus substitute stims. These findings complement both results from qualitative studies and comments from the autistic community promoting stimming as having beneficial effects and raising awareness of the need for greater understanding of such behaviours from the wider community (Kapp et al., 2019).

Results from this study should be interpreted while considering certain limitations. Participants were recruited through online support groups and promotion of the study on social media. As a result, participants reported that they had an autism diagnosis (providing the date of diagnosis), identified as or suspected that they were autistic, or were not autistic. Although autistic trait measures suggest that the group classifications (diagnosed, undiagnosed, not autistic) are accurate, some participants in each group may be misclassified using these criteria. Participants were also required to complete the questionnaires independently. Therefore the sample is unlikely to be representative of the autism population as a whole. Furthermore, the sample is largely female with few male and non-binary participants. Although the intention of the study was to include all genders, recruitment included a support group for autistic women, and this may have been a reason for the high number of female participants. The study included a small number of non-binary individuals, although the number was proportionally high in relation to frequency in the general population. Although the sample is not representative of the gender distribution usually observed in autism, it does include two under-studied groups, females and non-binary people. The analyses were repeated for females only and show the same pattern of results. A further advantage of the sample is that it includes a wide age-range. Although correlations with age were modest, in the diagnosed autistic group only older age was associated with fewer repetitive sensory motor behaviours and less hyposensitivity. This suggests that there are relatively few associations between age and repetitive behaviours and sensory sensitivity, although it is important to note that “change” within individuals was not measured in this study.

A further limitation of online studies in general is that a formal autism diagnosis cannot be confirmed. This study asked people to identify as having (1) an autism diagnosis, (2) identifying as autistic or seeking an autism diagnosis without currently having a diagnosis or (3) having no autism diagnosis or suspicion of being autistic. Only undiagnosed autistic people completed an autism screening measures, and all individuals scored above the cut-off for suspected autism. We cannot be certain that the diagnosed autistic group would meet criteria for the autism cut-off, or that the non-autistic group would not. However, the diagnosed and undiagnosed autistic groups show very similar scores across all measures and these are significantly different to the non-autistic group, providing some confidence in the group assignments. This study benefits from including a large sample of adults across a wide age-range.

These results show that repetitive behaviours and sensory sensitivities are higher in autistic than non-autistic adults, suggesting that these experiences continue into adulthood. All three groups demonstrate strong associations between both repetitive sensory motor behaviours and insistence on sameness with sensory hyper- and hypo-sensitivities. Stimming had a significant positive impact on autistic adults’ self-rated self-efficacy, and altering preferred stims reduced their efficacy. Results contradict the negative view of stimming often expressed in narratives of childhood, which suggest that stimming should be eradicated (see the following studies aiming to ‘treat’ repetitive behaviours in children Foxx & Azrin, 1973; Ventola et al., 2016; and adults O’Connor et al., 2018). Results of this study demonstrate that stimming has a positive effect and supports autistic individuals’ ability to cope with difficulties. Results do not negate the potential negative effects self-injurious or other stims could have on an individual particularly over time. The causes and impact of stimming and repetitive behaviours is not yet fully understood. Further research is needed to better understand effects of stimming, and how they may be utilised to support coping in everyday life.

## References

[CR1] American Psychiatric Association. (2013). *Diagnostic and statistical manual of mental disorders: Vol. 5th Edition*. American Psychiatric Publishing.

[CR2] Barrett, S. L., Uljarevic, M., Jones, C. R. G., & Leekam, S. R. (2018). Assessing subtypes of restricted and repetitive behaviour using the adult repetitive Behaviour Questionnaire-2 in autistic adults. *Molecular Autism*, *9*(1), 58. 30505424 10.1186/s13229-018-0242-4 PMC6258483

[CR3] Bradley, L., Shaw, R., Baron-Cohen, S., & Cassidy, S. (2021). Autistic adults’ Experiences of Camouflaging and its perceived impact on Mental Health. *Autism in Adulthood*, *3*(4), 320–329. 10.1089/aut.2020.0071 36601637 10.1089/aut.2020.0071 PMC8992917

[CR4] Burke, S. L., Li, T., Grudzien, A., & Garcia, S. (2021). Brief report: Improving employment interview self-efficacy among adults with autism and other Developmental Disabilities using virtual interactive training agents (ViTA). *Journal of Autism and Developmental Disorders*, *51*(2), 741–748. 10.1007/s10803-020-04571-8 32642959 10.1007/s10803-020-04571-8

[CR5] Charlton, R. A., Entecott, T., Belova, E., & Nwaordu, G. (2021). It feels like holding back something you need to say: Autistic and non-autistic adults accounts of sensory experiences and stimming. *Research in Autism Spectrum Disorders*, *89*, 101864. 10.1016/j.rasd.2021.101864

[CR6] Chowdhury, M., Benson, B. A., & Hillier, A. (2010). Changes in restricted repetitive behaviors with age: A study of high-functioning adults with Autism Spectrum Disorders. *Research in Autism Spectrum Disorders*, *4*(2), 210–216.

[CR7] Cook, J., Crane, L., Hull, L., Bourne, L., & Mandy, W. (2021). Self-reported camouflaging behaviours used by autistic adults during everyday social interactions. *Autism*, 13623613211026754. 10.1177/13623613211026754 10.1177/13623613211026754 PMC8814950 34180249

[CR8] Dachez, J., & Ndobo, A. (2018). Coping strategies of adults with high-functioning autism: A qualitative analysis. *Journal of Adult Development*, *25*(2), 86–95.

[CR9] Dammeyer, J. (2014). Symptoms of Autism among children with congenital deafblindness. *Journal of Autism and Developmental Disorders*, *44*(5), 1095–1102. 10.1007/s10803-013-1967-8 24127166 10.1007/s10803-013-1967-8

[CR10] Foxx, R. M., & Azrin, N. H. (1973). The elimination of autistic self-stimulatory behavior by overcorrection. *Journal of Applied Behavior Analysis*, *6*(1), 1–14. 10.1901/jaba.1973.6-1 16795380 10.1901/jaba.1973.6-1 PMC1310802

[CR11] Haigh, S. M. (2018). Variable sensory perception in autism. *European Journal of Neuroscience*, *47*(6), 602–609. 10.1111/ejn.13601 28474794 10.1111/ejn.13601

[CR12] Harrop, C., McConachie, H., Emsley, R., Leadbitter, K., Green, J., & The PACT Consortium. (2014). Restricted and repetitive behaviors in Autism Spectrum Disorders and typical Development: Cross-sectional and longitudinal comparisons. *Journal of Autism and Developmental Disorders*, *44*(5), 1207–1219. 24234675 10.1007/s10803-013-1986-5

[CR13] Hartshorne, T. S., Grialou, T. L., & Parker, K. R. (2005). Autistic-like behavior in CHARGE syndrome. *American Journal of Medical Genetics Part A*, *133A*(3), 257–261. 10.1002/ajmg.a.30545 15637726 10.1002/ajmg.a.30545

[CR14] Hirvikoski, T., & Blomqvist, M. (2014). High self-perceived stress and poor coping in intellectually able adults with autism spectrum disorder. *Autism*, *19*(6), 752–757. 10.1177/1362361314543530 25073750 10.1177/1362361314543530

[CR15] Hull, L., Petrides, K. V., Allison, C., Smith, P., Baron-Cohen, S., Lai, M. C., & Mandy, W. (2017). Putting on my best normal’: Social camouflaging in adults with Autism Spectrum Conditions. *Journal of Autism and Developmental Disorders*, *47*(8), 2519–2534. 28527095 10.1007/s10803-017-3166-5 PMC5509825

[CR16] Hwang, Y. I., Arnold, S., Srasuebkul, P., & Trollor, J. (2020). Understanding anxiety in adults on the autism spectrum: An investigation of its relationship with intolerance of uncertainty, sensory sensitivities and repetitive behaviours. *Autism*, *24*(2), 411–422. 10.1177/1362361319868907 31416327 10.1177/1362361319868907

[CR17] Ivy, S. E., & Ledford, J. R. (2022). A systematic review of behavioral interventions to reduce restricted or repetitive behavior of individuals with visual impairment. *Journal of Behavioral Education*, *31*(1), 94–122. 10.1007/s10864-020-09418-x

[CR18] Joyce, C., Honey, E., Leekam, S. R., Barrett, S. L., & Rodgers, J. (2017). Anxiety, intolerance of uncertainty and restricted and repetitive Behaviour: Insights directly from Young People with ASD. *Journal of Autism and Developmental Disorders*, *47*(12), 3789–3802. 28238024 10.1007/s10803-017-3027-2

[CR19] Kapp, S. K., Steward, R., Crane, L., Elliott, D., Elphick, C., Pellicano, E., & Russell, G. (2019). People should be allowed to do what they like’: Autistic adults’ views and experiences of stimming. *Autism*, *23*(7), 1782–1792. 10.1177/1362361319829628 30818970 10.1177/1362361319829628 PMC6728747

[CR20] Kargas, N., Lopez, B., Reddy, V., & Morris, P. (2015). The Relationship between Auditory Processing and Restricted, repetitive behaviors in adults with Autism Spectrum Disorders. *Journal of Autism and Developmental Disorders*, *45*(3), 658–668. 25178987 10.1007/s10803-014-2219-2

[CR21] Leekam, S. R., Prior, M. R., & Uljarevic, M. (2011). Restricted and repetitive behaviors in autism spectrum disorders: A review of research in the last decade. *Psychological Bulletin*, *137*(4), 562–593. 21574682 10.1037/a0023341

[CR22] Livingston, L. A., Colvert, E., Bolton, P., & Happe, F. (2018). Good social skills despite poor theory of mind: Exploring compensation in autism spectrum disorder. *Journal of Child Psychology and Psychiatry*, *0*(0), 10.1111/jcpp.12886 10.1111/jcpp.12886 PMC6334505 29582425

[CR23] MacLennan, K., O’Brien, S., & Tavassoli, T. (2022). In our own words: The Complex sensory experiences of autistic adults. *Journal of Autism and Developmental Disorders*, *52*(7), 3061–3075. 10.1007/s10803-021-05186-3 34255236 10.1007/s10803-021-05186-3 PMC9213348

[CR24] Manor-Binyamini, I., & Schreiber-Divon, M. (2019). Repetitive behaviors: Listening to the voice of people with high-functioning autism spectrum disorder. *Research in Autism Spectrum Disorders*, *64*, 23–30. 10.1016/j.rasd.2019.04.001

[CR25] Moore, H. L., Brice, S., Powell, L., Ingham, B., Freeston, M., Parr, J. R., & Rodgers, J. (2022). The Mediating Effects of Alexithymia, intolerance of uncertainty, and anxiety on the relationship between sensory Processing differences and restricted and repetitive Behaviours in autistic adults. *Journal of Autism and Developmental Disorders*, *52*(10), 4384–4396. 10.1007/s10803-021-05312-1 34643864 10.1007/s10803-021-05312-1 PMC9508023

[CR26] O’Connor, K., Lavoie, M., Desaulniers, B., & Audet, J. S. (2018). Cognitive psychophysiological treatment of bodily-focused repetitive behaviors in adults: An open trial. *Journal of Clinical Psychology*, *74*(3), 273–285. 10.1002/jclp.22501 28815684 10.1002/jclp.22501

[CR27] Ritvo, R., Ritvo, E., Guthrie, D., Ritvo, M., Hufnagel, D., McMahon, W., Tonge, B., Mataix-Cols, D., Jassi, A., Attwood, T., & Eloff, J. (2011). The Ritvo Autism Asperger Diagnostic Scale-Revised (RAADS-R): A scale to assist the diagnosis of Autism Spectrum disorder in adults: An International Validation Study. *Journal of Autism and Developmental Disorders*, *41*(8), 1076–1089. 21086033 10.1007/s10803-010-1133-5 PMC3134766

[CR28] Robertson, A. E., & Simmons, D. R. (2013). The relationship between sensory sensitivity and autistic traits in the General Population. *Journal of Autism and Developmental Disorders*, *43*(4), 775–784. 22832890 10.1007/s10803-012-1608-7

[CR29] Robertson, A. E., & Simmons, D. R. (2015). The sensory experiences of adults with Autism Spectrum disorder: A qualitative analysis. *Perception*, *44*(5), 569–586. 10.1068/p7833 26422904 10.1068/p7833

[CR30] Schulz, S. E., & Stevenson, R. A. (2018). Sensory hypersensitivity predicts repetitive behaviours in autistic and typically-developing children. *Autism*, *23*(4), 1028–1041. 10.1177/1362361318774559 30244585 10.1177/1362361318774559

[CR31] Schwartzer, R., & Jerusalem, M. (1995). Generalized self-efficacy scale. In J. Weinman, S. Wright, & M. Johnston (Eds.), *Measures in health psychology: A user’s profolio. Causal and control beliefs* (pp. 35–37). NFER-NELSON.

[CR32] Shattuck, P. T., Steinberg, J., Yu, J., Wei, X., Cooper, B. P., Newman, L., & Roux, A. M. (2014). Disability Identification and Self-Efficacy among College Students on the Autism Spectrum. *Autism Research and Treatment*, *2014*, 924182. 10.1155/2014/924182 PMC3953486 24707401

[CR33] South, M., Ozonoff, S., & McMahon, W. M. (2005). Repetitive behavior profiles in Asperger Syndrome and High-Functioning Autism. *Journal of Autism and Developmental Disorders*, *35*(2), 145–158. 15909401 10.1007/s10803-004-1992-8

[CR34] Stewart, R. L. (2015). Repetitive stereotyped behaviour or ‘stimming’: An online survey of 100 people on the autism spectrum. *Presented at the 2015 International Meeting for Autism Research* International Meeting for Autism Research. https://insar.confex.com/insar/2015/webprogram/Paper20115.html

[CR35] Ventola, P. E., Yang, D., Abdullahi, S. M., Paisley, C. A., Braconnier, M. L., & Sukhodolsky, D. G. (2016). Brief report: Reduced restricted and repetitive behaviors after pivotal response treatment. *Journal of Autism and Developmental Disorders*, *46*(8), 2813–2820. 10.1007/s10803-016-2813-6 27230762 10.1007/s10803-016-2813-6 PMC4939123

[CR36] Ward, D. M., & Esposito, M. C. K. (2019). Virtual reality in transition program for adults with autism: Self-Efficacy, confidence, and interview skills. *Contemporary School Psychology*, *23*(4), 423–431. 10.1007/s40688-018-0195-9

